# Supplementary material for: The effect of COVID certificates on vaccine uptake, health outcomes, and the economy
Source: Nat Commun. 2022 Jul 8;13:3942. doi: 10.1038/s41467-022-31394-1 (PMC9263819; doi:10.1038/s41467-022-31394-1)
Supplement: Supplementary file 1 — Supplementary Information [file 41467_2022_31394_MOESM1_ESM.pdf]

Supplementary information

Supplementary Table 1: Demographic, health infrastructure and economic indicators for France, Germany, and Italy.

	France	Germany	Italy
Demographics: *Median age* (2015) in years[1]	41.2	45.9	45.9
Demographics: *Old-age dependency ratio* (2020); this is the ratio of the number of people older than 64 relative to the number of people in the working-age (15-64 years); data are shown as the proportion of dependents per 100 working-age population[2]	33.69%	33.70%	36.57%
Health infrastructure: *Health expenditure per capita* (2019) in international-$; the sum of public and private annual health expenditure per person[3]	5,493	6,739	3,998
Health infrastructure: *Percentage of population covered by health insurance* (2010-2011); estimate of health insurance coverage as a percentage of total population –coverage includes affiliated members of health insurance or estimation of the population having free access to health care services provided by the State[4]	99.9%	100%	100%
Economy: *GDP per capita* (2020) in international-$[5]	42,026	50,922	38,992
Economy: *Income inequality* (2010); shown is the Gini coefficient –higher values indicate higher level of inequality– for equivalised household income for market income & disposable income[6]	0.51 & 0.29	0.52 & 0.29	0.50 & 0.33

[1] UN Population Division, World Population Prospects, 2017 Revision, https://esa.un.org/unpd/wpp/Download/Standard/Population/
[2] World Development Indicators - World Bank (2021.07.30), http://data.worldbank.org/data-catalog/world-development-indicators
[3] WHO, Global Health Observatory (GHO), https://ghoapi.azureedge.net/api/
[4] Scheil-Adlung, Xenia (2014), Universal Health Protection: Progress to Date and the Way Forward, International Labour Organization. OECD.Stat
[5] World Development Indicators - World Bank (2021.07.30), https://datacatalog.worldbank.org/search/dataset/0037712
[6] Incomes across the Distribution Database, http://www.lisdatacenter.org

Supplementary Table 2: COVID certificates: announcement date and regulations for France, Germany, and Italy.

Country	Announcement	Places where COVID certificates are required
France	12 July 2021[7]	Places of entertainment and leisure (e.g., cinemas, festivals, museums, sports, conferences, game rooms, amusement parks, cruise ships), places of social gathering (e.g., bars, cafés, restaurants, clubs), interregional public transport (e.g., domestic flights, trains, and coaches), and department stores and shopping centres with a surface greater than 20,000 square metres.[8]
Germany	10 August 2021[9]	Mandatory in areas with over 35 COVID cases per 100,000 inhabitants per week. Health care places (e.g., hospitals, care homes), hospitality venues (cafés, restaurants, etc.), events, indoor parties, and sports, and for the use of body-related services (e.g., hairdresser, massages, etc.).[10]
Italy	22 July 2021[11]	Places of entertainment and leisure (e.g., cinemas, festivals, museums, indoor sports, conferences, game rooms, amusement parks, cruise ships), places of social gathering (e.g., hospitality venues, bars, cafés, restaurants, clubs), interregional public transport (e.g., airports, train stations).[12]

The extension to workplaces was announced on 15 October 2021 in Italy[13] and on 23 November 2021 in Germany.[14]

7 Macron E. Adresse aux Français - 12 juillet 2021. https://www.elysee.fr/emmanuel-macron/2021/07/12/adresse-aux-francais-12-juillet-2021
8 https://www.covidpasscertificate.com/france-covid-pass-reopen-vaccinated-tourists/
9 Die Bundesregierung. Videoschaltkonferenz der Bundeskanzlerin mit den Regierungschefinnen und Regierungschefs der Länder am 10. August 2021. https://www.bundesregierung.de/resource/blob/974430/1949532/d3f1da493b643492b6313e8e6ac64966/2021-08-10-mpk-data.pdf?download=1
10 https://www.bundesregierung.de/breg-en/news/federal-regional-consultation-coronavirus-1949666
11 Presidenza del Consiglio dei Ministri. Comunicato stampa del Consiglio dei Ministri n. 30. 22 July 2021. https://www.governo.it/it/articolo/comunicato-stampa-del-consiglio-dei-ministri-n-30/17514
12 https://www.euronews.com/2021/07/23/italy-to-roll-out-covid-health-pass-for-bars-restaurants-and-museums
13 https://www.reuters.com/world/europe/italys-mandatory-covid-health-pass-work-sees-untroubled-launch-2021-10-15/
14 https://www.bundesgesundheitsministerium.de/ministerium/gesetze-und-verordnungen/guv-20-lp/ifsg-aend.html

Supplementary Table 3: Use of COVID certificates in OECD and EU countries (N/A = not before November 2021).

Country	Announcement date	Source
Australia	N/A	https://www.theguardian.com/australia-news/2021/oct/11/victoria-covid-update-vaccine-passports-trialled-as-pfizer-offered-to-all-age-groups
Austria	19/05/2021	https://www.thelocal.com/20210728/europe-how-does-use-of-health-passes-compare-in-europe-2/
Belgium	23/09/2021	https://www.rtbf.be/info/societe/detail_pass-sanitaire-au-restaurant-ou-dans-les-salles-de-sport-en-wallonie-des-avis-partages?id=10847152<br>https://www.adalovelaceinstitute.org/project/international-monitor-vaccine-passports-covid-status-apps/#belgium-10
Bulgaria	20/10/2021	https://www.euractiv.com/section/politics/short_news/bulgaria-introduces-green-covid-19-pass/
Canada	Various[ff]	https://globalnews.ca/news/8160105/canada-covid-vaccine-passports/
Chile	25/05/2021	https://www.efe.com/efe/espana/sociedad/chile-anuncia-un-pase-de-movilidad-que-otorga-mas-libertades-a-vacunados/10004-4543838
Colombia	25/07/2021	https://labsnews.com/en/notes/colombia-is-working-on-an-electronic-covid-19-vaccination-pass/
Costa Rica	13/10/2021	https://qcostarica.com/as-of-december-1-a-vaccination-certificate-will-be-mandatory-in-costa-rica/
Croatia	01/07/2021	https://www.garda.com/crisis24/news-alerts/496766/croatia-amendments-to-covid-19-countermeasures-will-be-implemented-from-july-1-update-28
Cyprus	09/07/2021	https://www.dw.com/en/cyprus-vaccine-drive-safepass-mandatory-no-more-free-covid-tests/a-58249253
Czech Republic	21/10/2021	https://www.expats.cz/czech-news/article/coronavirus-update-oct-21-2021<br>https://www.euractiv.com/section/politics/short_news/czechia-refuses-italian-way-says-no-to-obligatory-covid-pass-for-workers/<br>https://www.expats.cz/czech-news/article/coronavirus-update-oct-21-2021<br>https://www.expats.cz/czech-news/article/coronavirus-update-july-9-2021
Denmark	14/04/2021	https://www.healthcareitnews.com/news/emea/denmark-launches-covid-19-passport-coronapas
Estonia	26/08/2021	https://www.ecb.ee/news/new-coronavirus-restrictions-from-august-26/
Finland	06/08/2021	https://www.helsinkitimes.fi/finland/finland-news/domestic/19724-finnish-government-shows-green-light-to-coronavirus-pass.html
France	12/07/2021	https://www.elysee.fr/emmanuel-macron/2021/07/12/adresse-aux-francais-12-juillet-2021
Germany	10/08/2021	https://www.bundesregierung.de/breg-en/news/federal-regional-consultation-coronavirus-1949666
Greece	16/07/2021	https://www.reuters.com/world/europe/no-vaccines-no-dinner-indoor-greek-restaurants-accept-only-inoculated-customers-2021-07-16/
Hungary	N/A	https://www.euronews.com/travel/2021/10/12/green-pass-which-countries-in-europe-do-you-need-one-for
Iceland	N/A	https://www.euronews.com/travel/2021/10/12/green-pass-which-countries-in-europe-do-you-need-one-for
Ireland	29/06/2021	https://www.bbc.com/news/world-europe-57649546
Israel	07/03/2021	https://www.france24.com/en/middle-east/20210307-israel-opens-restaurants-and-bars-to-customers-vaccinated-against-covid-19
Italy	22/07/2021	https://www.governo.it/it/articolo/comunicato-stampa-del-consiglio-dei-ministri-n-30/17514
Japan	N/A	https://www.japantimes.co.jp/news/2021/12/14/national/japan-start-using-digital-vaccination-certificates-dec-20-via-smartphone-app/
Latvia	10/06/2021	https://www.laprensalatina.com/latvia-to-reopen-indoor-restaurants-to-vaccinated-people/
Lithuania	13/09/2021	https://www.roedl.com/insights/covid-19/lithuania-corona-covid-pass-vaccinated-national-certificate
Luxembourg	02/06/2021	https://www.wort.lu/fr/luxembourg/la-liberte-passera-par-le-covid-check-60b79a27de135b92362283bb
Malta	N/A	https://www.euronews.com/travel/2021/10/12/green-pass-which-countries-in-europe-do-you-need-one-for
Mexico	13/08/2021	https://www.covidpasscertificate.com/mexico-covid-passports/
Netherlands	16/11/2021	https://www.usnews.com/news/health-news/articles/2021-11-16/positive-virus-tests-reach-weekly-high-in-the-netherlands
New Zealand	N/A	https://www.reuters.com/world/asia-pacific/new-zealand-use-vaccine-certificates-delta-persists-2021-10-05/
Norway	N/A	https://www.euronews.com/travel/2021/10/12/green-pass-which-countries-in-europe-do-you-need-one-for
Poland	N/A	https://www.euronews.com/travel/2021/10/12/green-pass-which-countries-in-europe-do-you-need-one-for
Portugal	08/07/2021	https://www.lci.fr/sante/covid-19-le-portugal-elargit-l-usage-du-pass-sanitaire-aux-hotels-et-restaurants-2191149.html
Romania	17/09/2021	https://www.romania-insider.com/romania-green-pass-regulations-economy
Slovakia	17/07/2021	https://www.slovensko.sk/en/news/_digital-covid-pass
Slovenia	12/09/2021	https://www.total-slovenia-news.com/politics/8872-slovenia-tightens-covid-pass-restrictions
South Korea	01/12/2021	https://en.yna.co.kr/view/AEN20211213005851315

Spain	Various[15]	https://elpais.com/sociedad/2021-07-21/galicia-exigira-prueba-negativa-de-covid-o-certificado-de-vacunacion-para-acceder-al-interior-de-la-hosteleria-en-36-municipios.html
Sweden	09/12/2021	https://www.thelocal.se/20211209/swedens-new-vaccine-pass-plan-for-restaurants-and-long-distance-trains/
Switzerland	25/08/2021	https://www.thelocal.ch/20210825/breaking-switzerland-proposes-covid-certificates-indoors-in-bars-restaurants-and-gyms/
Turkey	06/09/2021	https://www.gov.uk/foreign-travel-advice/turkey/coronavirus
UK	08/12/2021	https://www.gov.uk/government/news/prime-minister-confirms-move-to-plan-b-in-england
USA	Various[ff]	https://www.covidpasscertificate.com/us-covid-passports/

Supplementary Figure 1. Ratio of second versus first vaccine doses around the date of the announcement of COVID certificates for France, Germany, and Italy.

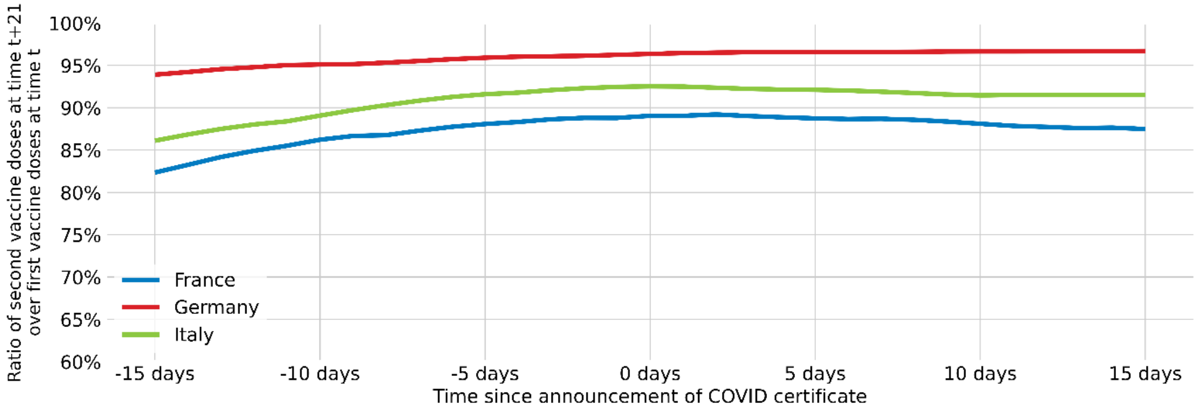

Supplementary Table 4. Distribution of the different types of vaccines in France, Germany, and Italy by 31 December 2021.

	France	Germany	Italy
BioNTech/Pfizer	79.5%	72.0%	70.1%
Moderna	11.7%	16.5%	18.2%
AstraZeneca	7.7%	8.4%	10.1%
Janssen Pharmaceutica NV	1.1%	3.1%	1.6%

Supplementary Table 5: Descriptive statistics of covariates to compute average effect of vaccination on economic activity.

	mean	std	min	25%	50%	75%	max
**Vaccinated people (per 100)**	17.8	26.1	0.0	0.0	0.0	33.7	89.5
**Cases**	150.8	217.4	0.0	12.4	64.1	204.3	2041.2
**Deaths**	2.5	3.7	0.0	0.2	0.9	3.4	28.3
**Reproduction rate**	1.0	0.4	0.0	0.9	1.0	1.2	5.5
**Mobility Index**	-20.9	17.1	-83.2	-30.4	-17.6	-8.5	14.5
**Stringency Index**	56.7	19.1	0.0	44.4	58.3	71.8	100.0

[15] By 22 September 2021, COVID certificates were in place in several parts of the country.

**Vaccination of partners**	14.5	18.0	0.0	0.0	2.3	31.5	68.0
**Deaths in partners**	1.7	1.5	0.0	0.5	1.2	2.3	9.1
**Cases in partners**	110.9	100.5	0.7	31.5	87.8	157.3	654.1
**GDP of partners**	-2.8	2.4	-16.5	-3.4	-2.2	-1.3	1.5

Supplementary Table 6: Regression results, alternative dependent variables.

Dependent variable	**Mobility Index**			**GDP**
	**Baseline**	**Controls (trade partners)**	**Direct effect**	**Direct effect**
Vaccinated people (per 100)	0.066***	0.069***	0.036**	0.028
	(0.033 , 0.099)	(0.037 , 0.101)	(0.004 , 0.068)	(-0.008 , 0.065)
Cases (lag 4)	-0.010***	-0.009***	-0.003***	
	(-0.012 , -0.008)	(-0.011 , -0.007)	(-0.005 , -0.001)	
Deaths (lag 4)	-0.281***	-0.199***	-0.039	
	(-0.394 , -0.168)	(-0.312 , -0.086)	(-0.159 , 0.080)	
Reproduction rate (lag 4)	-4.008***	-3.151***	-1.334***	
	(-4.836 , -3.181)	(-3.969 , -2.333)	(-2.154 , -0.514)	
Stringency Index (lag 4)	-0.168***	-0.162***	-0.172***	
	(-0.192 , -0.143)	(-0.186 , -0.139)	(-0.195 , -0.148)	
Temperature	0.457***	0.311***	0.268***	-0.004
	(0.388 , 0.526)	(0.244 , 0.379)	(0.202 , 0.335)	(-0.089 , 0.081)
Vaccination of trade partners		-0.314***	-0.303***	-0.022
		(-0.390 , -0.237)	(-0.378 , -0.229)	(-0.111 , 0.067)
Deaths in trade partners		-0.633***	-0.247	-0.055
		(-1.083 , -0.183)	(-0.700 , 0.206)	(-0.723 , 0.614)
Cases in partners		-0.005*	0.001	0.003
		(-0.011 , 0.002)	(-0.005 , 0.007)	(-0.007 , 0.014)
GDP of partners		3.574***	3.328***	0.296
		(3.216 , 3.932)	(2.974 , 3.682)	(-0.154 , 0.745)
Cases			-0.005***	0.000
			(-0.007 , -0.003)	(-0.002 , 0.003)
Deaths			-0.612***	-0.155*
			(-0.743 , -0.480)	(-0.321 , 0.011)
Reproduction rate			-0.925*	-1.164**
			(-1.948 , 0.098)	(-2.210 , -0.118)
Stringency Index				-0.043***
				(-0.075 , -0.010)
Country dummies	Yes	Yes	Yes	Yes
Week dummies	*Yes*	*Yes*	*Yes*	Yes
Observations	4230	4230	4230	355
R2	0.757	0.779	0.79	0.861
Adjusted R2	0.749	0.771	0.782	0.833
Residual Std. Error	8.489 (df=4086)	8.098 (df=4082)	7.909 (df=4079)	2.369 (df=293)
F Statistic	89.038*** (df=143; 4086)	97.969*** (df=147; 4082)	102.001*** (df=150; 4079)	29.871*** (df=61; 293)

* $p < 0.1$, ** $p < 0.05$, and ***$p < 0.01$
