# Supplementary material for: The effect of COVID certificates on vaccine uptake, health outcomes, and the economy
Source: Nat Commun. 2022 Jul 8;13:3942. doi: 10.1038/s41467-022-31394-1 (PMC9263819; doi:10.1038/s41467-022-31394-1)
Supplement: Supplementary file 3 — Reporting Summary [file 41467_2022_31394_MOESM3_ESM.pdf]

nature portfolio

Corresponding author(s): Miquel Oliu-Barton, Bary Pradelski, Nicolas Woloszko

Last updated by author(s): May 14, 2022

Reporting Summary

Nature Portfolio wishes to improve the reproducibility of the work that we publish. This form provides structure for consistency and transparency in reporting. For further information on Nature Portfolio policies, see our Editorial Policies and the Editorial Policy Checklist.

Statistics

For all statistical analyses, confirm that the following items are present in the figure legend, table legend, main text, or Methods section.

n/a	Confirmed	
☐	☒	The exact sample size (*n*) for each experimental group/condition, given as a discrete number and unit of measurement
☐	☒	A statement on whether measurements were taken from distinct samples or whether the same sample was measured repeatedly
☐	☒	The statistical test(s) used AND whether they are one- or two-sided<br>*Only common tests should be described solely by name; describe more complex techniques in the Methods section.*
☐	☒	A description of all covariates tested
☐	☒	A description of any assumptions or corrections, such as tests of normality and adjustment for multiple comparisons
☐	☒	A full description of the statistical parameters including central tendency (e.g. means) or other basic estimates (e.g. regression coefficient) AND variation (e.g. standard deviation) or associated estimates of uncertainty (e.g. confidence intervals)
☐	☒	For null hypothesis testing, the test statistic (e.g. *F*, *t*, *r*) with confidence intervals, effect sizes, degrees of freedom and *P* value noted<br>*Give P values as exact values whenever suitable.*
☒	☐	For Bayesian analysis, information on the choice of priors and Markov chain Monte Carlo settings
☒	☐	For hierarchical and complex designs, identification of the appropriate level for tests and full reporting of outcomes
☒	☐	Estimates of effect sizes (e.g. Cohen's *d*, Pearson's *r*), indicating how they were calculated

Our web collection on statistics for biologists contains articles on many of the points above.

Software and code

Policy information about availability of computer code

Data collection

For all OECD and EU countries, the share of the population who received one dose of a COVID-19 vaccine, two doses of a COVID-19 vaccine, hospital admissions per 1 million, daily ICU patients per 1 million, daily deaths per 1 million, and population estimates have been retrieved from Our World In Data.46 For France age-stratified data on hospital admissions, ICU patients, and deaths was retrieved from official government sources (https://www.data.gouv.fr/fr/datasets/synthese-des-indicateurs-de-suivi-de-lepidemie-covid-19/) and deaths outside hospitals from the French Institute for Demographic Studies (INED) (https://dc-covid.site.ined.fr/en/data/france/). For Italy age-stratified data on deaths was also retrieved from INED (https://dc-covid.site.ined.fr/en/data/italy/).

Age-stratified vaccine uptake statistics for France and Italy were both retrieved from the European Centre of Disease Prevention and Control (note that such data is not available for Germany) (https://vaccinetracker.ecdc.europa.eu/public/extensions/COVID-19/vaccine-tracker.html#age-group-tab).
The share of different vaccines used until the end of 2021 in France, Germany, and Italy (made by BioNTech/Pfizer, Moderna, AstraZeneca, Janssen Pharmaceutica NV) have been retrieved from the official government sources (France: https://covidtracker.fr/vaccintracker/, Germany: https://impfdashboard.de/, Italy: https://www.governo.it/it/cscovid19/report-vaccini/).

The OECD Weekly Tracker (citation 40)

Vaccine acceptance. Is taken from the University of Maryland Social Data Science Center "Global COVID-19 Trends and Impact Survey" in partnership with Facebook. (https://jpsm.umd.edu/research/global-covid-19-trends-and-impact-survey%2C-partnership-facebook)

A mobility index is built from the Google Mobility reports (https://www.google.com/covid19/mobility/),

Temperature. Daily temperature series for the 46 OECD and G20 countries across 2020 and 2021 were collected from the National Oceanic and Atmospheric Administration's National Centers for Environmental Information (https://www.ncei.noaa.gov/). The Global Historical

Climatology Network daily (GHCNd) provides daily climate summaries from land surface stations across the globe. Temperature data for each station were averaged at the country level.

Policy interventions. For each country, we consider the date when COVID certificates for day-to-day use were announced, namely, 12 July 2021 for France, 10 August 2021 for Germany, and 22 July 2021 for Italy. See Table A2 (Appendix) for details of the corresponding regulations.

OECD and EU countries that announced the use of COVID certificates before 22 September 2021. Austria, Canada, Chile, Colombia, Croatia, Cyprus, Denmark, Estonia, Finland, France, Germany, Greece, Ireland, Israel, Italy, Latvia, Lithuania, Luxembourg, Mexico, Portugal, Romania, Slovakia, Slovenia, Spain, Switzerland, the United States.
Remaining OECD and EU countries. Australia, Belgium, Bulgaria, Costa Rica, Czech Republic, Hungary, Iceland, Japan, Malta, the Netherlands, New Zealand, Norway, Poland, South Korea, Sweden, Turkey, the United Kingdom.
Donor pool countries for synthetic control. Australia, Belgium, Czech Republic, Hungary, Japan, Malta, the Netherlands, New Zealand, Norway, Poland, South Korea, Sweden, Turkey, the United Kingdom. (Costa Rica, and Iceland have been removed from the donor pool used for the synthetic control method due to lack of data for covariates. Bulgaria has been removed from the donor pool due to lack of vaccination coverage data over most of the analysed period.)
See Table A3 (Appendix) for information on all OECD and EU countries regarding the implementation of COVID certificates.

Data analysis: Excel, Python (packages: curve_fit, scipy.optimize)

For manuscripts utilizing custom algorithms or software that are central to the research but not yet described in published literature, software must be made available to editors and reviewers. We strongly encourage code deposition in a community repository (e.g. GitHub). See the Nature Portfolio guidelines for submitting code & software for further information.

Data

Policy information about availability of data

All manuscripts must include a data availability statement. This statement should provide the following information, where applicable:

- Accession codes, unique identifiers, or web links for publicly available datasets
- A description of any restrictions on data availability
- For clinical datasets or third party data, please ensure that the statement adheres to our policy

All data in the study is publicly available as detailed above with all sources (see Data collection).

Field-specific reporting

Please select the one below that is the best fit for your research. If you are not sure, read the appropriate sections before making your selection.

☐ Life sciences ☒ Behavioural & social sciences ☐ Ecological, evolutionary & environmental sciences

For a reference copy of the document with all sections, see nature.com/documents/nr-reporting-summary-flat.pdf

Behavioural & social sciences study design

All studies must disclose on these points even when the disclosure is negative.

Study description	The study is using mainly quantitative data and analysis (e.g., vaccine uptake, deaths, GDP). The qualitative element is the interpretation of various non-pharmceutical interventions and whether are classified as COVID certificates or not.
Research sample	The research sample concerns all EU and OECD countries (vaccine uptake) as well as health and economic data for France, Germany, and Italy. See data collection above for details.
Sampling strategy	Samples are the whole population of each respective country, and, when applicable, stratified by over 60 years old and under 60 years old.
Data collection	The data was collected from public sources. There were no experimental treatments.
Timing	All data were retrieved in the first week of January 2022.
Data exclusions	No data was excluded from the analysis.
Non-participation	There were no participants involved in the study. It was purely observational based on publicly available data.
Randomization	Randomization was not applicable as there was no A/B testing. The results rely on counterfactual construction via theoretical and statistical modelling approaches.

Reporting for specific materials, systems and methods

We require information from authors about some types of materials, experimental systems and methods used in many studies. Here, indicate whether each material, system or method listed is relevant to your study. If you are not sure if a list item applies to your research, read the appropriate section before selecting a response.

Materials & experimental systems

n/a	Involved in the study
☒	☐ Antibodies
☒	☐ Eukaryotic cell lines
☒	☐ Palaeontology and archaeology
☒	☐ Animals and other organisms
☒	☐ Human research participants
☒	☐ Clinical data
☒	☐ Dual use research of concern

Methods

n/a	Involved in the study
☒	☐ ChIP-seq
☒	☐ Flow cytometry
☒	☐ MRI-based neuroimaging
